# THRESHER: A tool for evidence-based, phylogeny-corrected SNP thresholds for whole genome molecular epidemiology

**DOI:** 10.64898/2026.10.07.26364951

**Published:** 2026-10-11

**Authors:** Qianxuan She, Andries Feder, Erin Theiller, Joseph P. Zackular, Ahmed M. Moustafa, Paul J. Planet

## Abstract

Whole-genome sequencing (WGS) is the gold standard for bacterial pathogen surveillance, where transmission is commonly inferred by calculating pairwise single nucleotide polymorphisms (SNPs) and applying a fixed threshold below which two genomes are considered the “same” strain. Such cutoffs are largely arbitrary. The relationship between genetic distance and epidemiological linkage varies with sample size, sampling duration, setting, and patterns of infection and carriage. A threshold calibrated in one study may not transfer to another. To address this, we present THRESHER, a pipeline that infers data-driven phylothresholds directly from each dataset’s genomic structure. THRESHER implements four endpoint methods, *plateau, public, peak* , and *discrepancy* , with each reflecting a distinct hypothesis about what constitutes the “sameness” of a strain. Across 55 published datasets spanning *Staphylococcus aureus, Clostridioides difficile* , and *Klebsiella pneumoniae* , THRESHER reproduced the originally reported strain compositions in smaller studies. In larger studies, where the two diverged, SNP distances and phylogenetic topology supported THRESHER’s assignments. In simulated populations with known strain structure, THRESHER recovered transmission clusters with substantially higher sensitivity than fixed cutoffs while retaining high specificity.

## Full Text Availability

The license terms selected by the author(s) for this preprint version do not permit archiving in PMC. The full text is available from the preprint server.

